# Lipid Biomarkers for Amyotrophic Lateral Sclerosis

**DOI:** 10.3389/fneur.2019.00284

**Published:** 2019-04-04

**Authors:** Jose-Luis González De Aguilar

**Affiliations:** ^1^Université de Strasbourg, UMR_S1118, Strasbourg, France; ^2^INSERM, U1118, Mécanismes Centraux et Périphériques de la Neurodégénerescence, Strasbourg, France

**Keywords:** amyotrophic lateral sclerosis, biomarker, cholesterol, fatty acid, triglyceride

## Abstract

Amyotrophic lateral sclerosis (ALS) is a fatal degenerative disease primarily characterized by the selective loss of upper and lower motor neurons. To date, there is still an unmet need for robust and practical biomarkers that could estimate the risk of the disease and its progression. Based on metabolic modifications observed at the level of the whole body, different classes of lipids have been proposed as potential biomarkers. This review summarizes investigations carried out over the last decade that focused on changes in three major lipid species, namely cholesterol, triglycerides and fatty acids. Despite some contradictory findings, it is becoming increasingly accepted that dyslipidemia, and related aberrant energy homeostasis, must be considered as essential components of the pathological process. Therefore, it is tempting to envisage dietary interventions as a means to counterbalance the metabolic disturbances and ameliorate the patient's quality of life.

## ALS and the Need for Biomarkers

Amyotrophic lateral sclerosis (ALS) is a degenerative disease of upper and lower motor neurons mainly characterized by progressive muscle wasting, fasciculations, dysarthria, dysphagia, altered reflexes, and spasticity. It affects about 2 per 100,000 people per year, and usually appears at 40–70 years of age. A significant proportion of cases also presents cognitive or behavioral abnormalities typical of frontotemporal dementia (FTD). The etiology of ALS still remains elusive. About 90% of cases are considered as sporadic. The remaining 10% are inherited mostly in an autosomal dominant manner. Most familial cases can be explained by mutations in four major genes, including *C9ORF72, SOD1, FUS*, and *TARDBP*. Based on this genetic diversity, multiple pathogenic mechanisms have been implicated in triggering motor neuron degeneration, adding considerable complexity to the understanding of the disease (1).

From a clinical point of view, ALS is easily recognized in its full-blown presentation. However, the diagnostic process may be challenging at very early stages. The diagnosis is based on clinical examination, electrophysiological findings, medical history, and exclusion of confounding disorders. In practice, a correct diagnosis may take as long as 1 year (2). Moreover, disease progression is very heterogeneous. Death may occur between 1 and 5 years after diagnosis, but 20% of patients live longer than 5 years, and 10% survive for more than 10 years (3). Promising biomarkers of diagnosis and prognosis have been proposed based on advanced neurophysiological and neuroimaging techniques. However, many of these practices still lack validation and standardization between clinical centers, and they have been applied only to small cohorts of patients [(4–6), and references therein].

As far as molecular biomarkers are concerned, a great number of molecules have been isolated from patient material, including cerebrospinal fluid, blood and tissues, that recognized and/or monitored ALS with more or less accuracy [(7, 8), and references therein]. To date, changes in the amounts of neurofilament proteins found in cerebrospinal fluid and blood have been postulated as the most promising candidates [(9), and references therein]. However, these and other proposed molecular markers have not reached routine clinical application. Therefore, there is still an incontestable lack of robust and practical biomarkers that could facilitate an earlier diagnosis and improve the prognosis of ALS.

## Altered Energy Homeostasis and Lipid Metabolism in ALS

Amyotrophic lateral sclerosis was classically attributed to an intrinsic defect of upper and lower motor neurons. Now it is generally accepted that non-neuronal cells surrounding motor neurons, additional neuronal cell types, as well as other cells outside the nervous system participate actively in the pathological process [(10–12), and references therein]. In particular, compelling evidence has emerged over the last decade showing a characteristic imbalance between energy intake and consumption, which is associated with metabolic alterations at the level of the whole body of yet unexplained etiology [(13), and references therein]. Seminal studies revealed that many ALS patients show an increase in energy expenditure, or hypermetabolism, which could account, at least in part, for the decline of their nutritional status (14, 15). It was also recently reported that hypermetabolic patients have a worse prognosis than normometabolic ones (16), which could be related to a detrimental weight loss. In fact, patients that lost more than 5% of body mass at the time of diagnosis had an increased risk of death (17). In addition, a lower body mass index appeared to precede the symptomatic stage of the disease (18). Overall, these studies strongly support that the energy imbalance in ALS could contribute to the rapid deterioration of the patients.

The origin of the hypermetabolism in ALS is currently unknown, although recent studies have pointed to the altered function of hypothalamic neurons involved in the regulation of food intake and energy homeostasis (19, 20). From a therapeutic point of view, pioneering preclinical studies conducted on an ALS mouse model, which carries a mutation in the *Sod1* gene, provided part of the answer to this question. An increase in energy consumption occurs in these mice well-before the onset of the first motor symptoms. This is accompanied by a reduction of adiposity and lower levels of circulating leptin. Most importantly, these studies revealed that sustaining the hypermetabolic rate of ALS mice with a highly-energetic high-fat diet partially protected motor neurons and extended lifespan (21). Likewise, a higher premorbid intake of high-fat food was observed in ALS patients (18). Moreover, a moderate increase in fat mass over the course of the disease was associated with a decreased risk of death, and increasing circulating levels of leptin were positively associated with longer survival (17, 22). Although there is no conclusive evidence of a mechanistic link between the hypermetabolism present in ALS and altered levels of lipids, altogether, these studies suggest that the utilization of lipids as energy substrates could offer benefit, by counteracting an increased metabolic rate and compensating the associated weight loss. In this respect, several pilot studies reported positive effects of highly caloric fat supplements on ALS patients (23, 24). It was also shown that the administration of acetyl-L-carnitine, which supports the transport of fatty acids into mitochondria for being used as energy substrates, retarded the worsening of the patients (25). Recent research has made efforts to identify specific changes in lipid metabolism that could provide clues for future nutritional interventions, as well as serve as robust biomarkers for the disease. This review covers some of the most significant findings published during the last decade.

## Apolipoprotein E and the Risk of ALS

Apolipoprotein E (APOE) is a constituent of lipoprotein particles primarily involved in the transport of triglycerides and their clearance from the bloodstream. It is mainly synthesized in the liver but it is also produced by astrocytes in the brain, where APOE is the most important cholesterol carrier. The human *APOE* gene exists as three major alleles called ε2, ε3, and ε4. The identification of *APOE* ε4 as a risk factor for Alzheimer's disease represented a major breakthrough in the field [(26), and references therein]. On the contrary, most studies on ALS did not observe any association of *APOE* ε4 with an increased risk (27–30), excepted some recent findings (31). Additional reports showed complex interactions between particular *APOE* alleles and other genetic or physiopathological variables. Penco and collaborators identified a combination of seven genetic variants, inluding one affecting *APOE*, that distinguished between ALS patients and control subjects (32). It was also found that individuals who had suffered from head trauma in the adulthood were more prone to have ALS, and this association was stronger in the presence of *APOE* ε4 (33). In contrast, the frequency of *APOE* ε2, which is *a priori* neuroprotective, was higher in ALS patients that had practiced sport regularly (30). *APOE* ε2 also increased the risk of developing FTD in a cohort of patients with ALS (34). It must be noted, however, that the implication of *APOE* in the incidence of this form of dementia is rather controversial. The increase in the probability of having FTD was associated with *APOE* ε2 in some cases, and with *APOE* ε4 in other cases [(35), and references therein].

The influence of *APOE* on the course of ALS has also been contradictory. Initial reports revealed that *APOE* ε4 was associated with earlier age at onset but not with disease duration (27). However, follow-up studies failed to show any relationship between *APOE* ε4 and age at onset or rate of progression, although this allele was more frequent in men with bulbar-onset ALS (28). Parallel investigations did not find any association between the *APOE* genotype and age of onset, site of onset, rate of progression, cognitive impairment or survival (36). Overall, the implication of *APOE* in the incidence and progression of ALS is therefore not clearly established.

## The Intriguing Case of Cholesterol

Cholesterol is an essential lipid molecule, which is transported through the bloodstream by several types of lipoprotein particles. In clinical practice, increased levels of total cholesterol or low-density lipoprotein cholesterol (LDL-c), in combination with decreased levels of high-density lipoprotein cholesterol (HDL-c), are indicative of a higher risk of atherosclerotic cardiovascular disease. In the case of ALS, hypercholesterolemia, as detected prior to the onset of motor symptoms, was initially associated with a lower risk (37). However, follow-up studies contradicted these findings. An increase in the premorbid intake of cholesterol was associated with a higher incidence of the disease, as shown after examination of dietary habits obtained from food frequency questionnaires (18). In addition, individuals with increased levels of LDL-c and a higher LDL-c/HDL-c ratio were more prone to develop ALS later (38). Finally, the analysis of GWAS databases revealed that particular alleles predisposing to elevated levels of LDL-c and total cholesterol appeared associated with an increased risk (39).

In many studies, the proportion of hypercholesterolemia individuals or the average contents of total cholesterol and LDL-c were shown to be higher in the ALS population and, in some cases, this increase was noticeable at the time of diagnosis (40–44). In agreement with these findings, a detailed analysis of circulating lipoprotein particles also showed increased levels of LDL-1, which is a LDL subfraction very enriched in cholesterol (44). Other reports, however, did not find clear-cut differences (45–48), or even revealed opposite results (49).

From a prognostic point of view, decreased levels of total cholesterol or LDL-c and a lower LDL-c/HDL-c ratio were associated with a severe respiratory impairment (42, 45). Contrasting with these findings, Delaye and collaborators did not observe any association between several cholesterol parameters and disease progression (44). Yet, most authors agree that hypercholesterolemia, present as elevated levels of total cholesterol and LDL-c or a higher LDL-c/HDL-c ratio, associates with longer survival. This association, however, did not reach significance after adjusting for potential confounding demographic and clinical factors (37, 40, 43, 49–51).

In addition to the biomarker potential of cholesterol *per se*, a few studies have focused on the implication of oxysterols, which are oxidized derivatives mainly involved in maintaining cholesterol homeostasis. Levels of several oxysterol metabolites, including 27-hydroxycholesterol, 24-hydroxycholesterol esters, and 3β,7α-dihydroxycholest-5-en-26-oic acid and other related compounds, were shown to be lower in ALS patients. These changes were detected in blood or cerebrospinal fluid, or both, and they were attributed to a deficit in the metabolism of excess cholesterol, which would result in subsequent toxicity in the brain (46, 52, 53). On the other hand, additional studies reported increased levels of 25-hydroxycholesterol in cerebrospinal fluid and serum of ALS patients. The accumulation of this toxic oxysterol derivative was associated, at least in serum, with a higher rate of disease progression (54).

## The Energizing Triglycerides in ALS

Triglycerides are a primary source of energy for the body but, when accumulated in an excessive manner, they represent an important risk factor for cardiovascular disease. Triglyceride contents should be expected to change in ALS patients according to their characteristic high rate of energy expenditure. In this respect, the proportion of hypertriglyceridemia individuals was more important among ALS patients than in the normal population (55). Hypertriglyceridemia was also found in ALS women (42), and higher triglyceride levels were associated with a better functional status (48). Other reports, however, failed to reproduce these findings (40, 47, 48). Moreover, Blasco and collaborators identified a lipidomic signature in the cerebrospinal fluid of ALS patients, in which certain triglyceride species were found reduced at levels associated with a better prognosis (56). Finally, as in the case of cholesterol, hypertriglyceridemia was associated with longer survival, but this association appeared to have no effect after adjusting for confounding factors (47, 55).

## The Entrance of Fatty Acids on Stage

Fatty acids are lipid molecules key for sustaining the structural integrity of cell membranes, providing energy and serving in signaling pathways. They can be mainly transported through the bloodstream attached to a glycerol molecule (that is, in the form of triglycerides) or as non-esterified free fatty acids. The studies relating to the implication of fatty acids as biomarkers for ALS are scarce. Based on food frequency questionnaires, Fitzgerald and collaborators showed that a higher intake of ω3 polyunsaturated fatty acids, which are considered as neuroprotective factors, were associated with a reduced risk of ALS (57). Similar studies did not find the same association but rather reported a higher premorbid intake of *trans*- and saturated fatty acids associated with an increased risk (18). On average, the proportion of polyunsaturated fatty acids in the lipid fraction of clotted blood was decreased in ALS patients while that of monounsaturated fatty acids was concomitantly increased (58). Polyunsaturated fatty acids were also lower in the free fatty acid fraction of plasma (59). Finally, a higher palmitoleic/palmitic fatty acid ratio, indicative of increased adiposity, correlated with a better functional status, and was associated with longer survival (58).

## Conclusion

Over the last decade, many lipid molecules have been proposed as promising biomarkers for ALS, but none of them has been translated into effective tools in clinical practice. There are several issues of concern that still need to be addressed. On the one hand, the etiology of ALS is multifactorial, and it is likely that the pathological process in subpopulations of patients, with different genetic and environmental backgrounds, is not the same. In the future, the use of cohorts of well-defined patients should improve statistical robustness. It would also be interesting to compare between patients with ALS and other patients suffering from mimic conditions. On the other hand, lipid changes at the level of the whole body can be affected by a myriad of factors, including genetic, nutritional, physical and pathological factors, which can introduce bias on the results. It is also noteworthy to mention that for those studies that used food frequency questionnaires to estimate food preferences and evaluate eating behavior, they depend, at least in part, on their interpretation probing the patient's perception of food intake, hence lacking sensitivity and objectiveness. Therefore, protocols and measurements need to be standardized between study centers.

Despite some conflicting findings, most studies presented in this review show important alterations of the circulating contents of cholesterol (and related lipoprotein particles), triglycerides and fatty acids, which occur prior to and over the course of ALS. These changes seem to reflect a metabolic environment, which would be appropriate to meet the high energy demands imposed by the increased metabolic rate present in the disease. The understanding of the mechanisms underlying this “low-grade dyslipidemia” is still insufficient but, from a clinical point of view, it leaves open the possibility for therapeutic nutritional intervention. In this respect, recent studies that analyzed the eating behavior of ALS patients revealed marked modifications in their food preferences. In particular, an increase in the intake of saturated fat and meat protein was associated with longer survival (60–62). Moreover, two clinical trials have been initiated, which aim at retarding disease progression by using high-caloric food supplements. The first trial (NCT02306590) is a randomized, parallel-group, double-blind study that compares between placebo and a treatment consisting of a high caloric fatty diet, which is equivalent to an additional intake of 45 g fat per day. The primary objective of this study is to evaluate the impact on survival. The second trial is a randomized, parallel-group, open label study that will determine the effects of a high-protein, high-energy supplement on the functional status of newly diagnosed ALS patients (NCT02152449). The results of these trials as well as the ongoing research on lipid biomarkers and on the understanding of their implication in ALS will certainly pave the way for developing new therapeutic tools.

## Author Contributions

The author confirms being the sole contributor of this work and has approved it for publication.

### Conflict of Interest Statement

The author declares that the research was conducted in the absence of any commercial or financial relationships that could be construed as a potential conflict of interest.
